# Skin Diseases in Donkeys and Mules—An Update

**DOI:** 10.3390/ani11010065

**Published:** 2020-12-31

**Authors:** Telma S. Lima, Raquel A. F. Silva, Raquel M. F. Pereira, Karoline L. Soares, Nayadjala T. A. Santos, Mônica S. Sousa, Fábio S. Mendonça, Ricardo B. Lucena

**Affiliations:** 1 Graduate Program in Veterinary Medicine, Federal Rural University of Pernambuco (UFRPE), Rua Dom Manuel de Medeiros, S/N Dois Irmãos, 52171-900 Recife-PE, Brazil; telma.lima@ufrpe.br (T.S.L.); raquel.mota@ufrpe.br (R.M.F.P.); karoline.lacerda@academico.ufpb.br (K.L.S.); nayadjala.santos@ufrpe.br (N.T.A.S.); fabio.mendonca@ufrpe.br (F.S.M.); 2 Graduate Program in Animal Science and Health, Rural Health and Technology Center (CSTR), Federal University of Campina Grande (UFCG), Avenida Universitária, S/N Jatobá, 58708-110 Patos-PB, Brazil; raquel.annes@estudante.ufcg.edu.br (R.A.F.S.); monica.shinneider@estudante.ufcg.edu.br (M.S.S.); 3 Graduate Program in Animal Science, Department of Agrarian Sciences (CCA), Federal University of Paraíba (UFPB), 12, Rod. PB-079, 58397-000 Areia-PB, Brazil

**Keywords:** skin, integument, donkey, mule

## Abstract

**Simple Summary:**

Equids are part of the history of many countries, including Brazil, where they were used in trade routes and expansion of the current states. Several skin diseases affect these animals; however, visibility is higher on horses than on donkeys and mules, which is linked to regional cultural and socioeconomic factors, even resulting in a decline of the world population of these animals. In this context, the objective of this study was to review which skin diseases have been reported in the scientific literature with emphasis on skin pathologies.

**Abstract:**

The skin of donkeys and mules represents a promising source of income; however, cultural, productive, and infectious factors can directly interfere with the quality of the integumentary tissue and well-being of these species. The objective of this study is to present a literature review on equine dermatopathies. This literature review included scientific articles related to equine medicine and breeding according to pre-established search terms and expressions published in recently articles. The evaluation of the clinical and pathological behavior of dermatopathies implies the use of control strategies and the recognition of pathological patterns that may be particular to the species.

## 1. Introduction

Donkeys and mules represent an important share of the world’s equids, totaling about 44 million in recent years, of which approximately 1 million are in Brazil [1,2]. The breeding of these animals is important in developing countries, and their introduction in the territories was quite variable, being associated with the colonial period in South America [3]. The work of donkeys and mules ranges from field work to recreation and entertainment [4], safeguarding socioeconomic importance in many states of Brazil, especially in eastern Brazil.

Many of the phenotypic and physiological characteristics of these animals favor sustainable breeding to increase regional economy. Donkey breeding, however, still presents major challenges in Brazil, which sees their population gradually decreasing over the years [5]. On the other hand, there is a trend toward expansion of the consumer market for meat, milk, and especially donkey skin, given the demand for these products in Asian countries such as China [6].

In this context, the investigation of diseases affecting donkeys and mules becomes important and gives rise to discussions on animal welfare and public health. This is demonstrated particularly in serological studies that show the participation of donkeys and mules in the epidemiological chain of reportable diseases [7,8]. It is furthermore important to highlight that cultural factors may influence the frequency of these diseases, affecting both animal and human health and welfare. Integrating these concepts within the population of an increasingly technological world is perhaps the greatest challenge of sustainable breeding of donkeys and mules today.

Regarding skin diseases in productive animals, the literature lacks studies on dermatopathies and their risk factors, especially in Brazil. Until recently, retrospective studies in ruminants and equids [9,10] have been highlighted in the country, whereas studies on donkeys are still incipient [11,12]. In this context, the objective of this study was to present a literature review on skin diseases in donkeys and mules.

To identify the main dermatopathies diagnosed in equids today, this study consists of a literature review containing the main studies on equine medicine and breeding, in which four major themes were initially proposed to direct the bibliographic research: (a) the use of donkeys and mules in Brazil and in the world; (b) the main diseases of donkeys and mules; (c) skin diseases diagnosed in donkeys and mules; and (d) equid dermatopathies in northeastern Brazil.

The search terms or expressions were chosen based on the major topics listed above and then typed into the major digital veterinary medicine libraries. Where necessary, Boolean operators such as and or not were used. The eligibility criteria were the terms and/or expressions, timeliness, status, and relevance of the publication according to the major themes. For this purpose, studies published or in press preferably from 2016 to 2020 were considered. The search was expanded to the last ten years when the terms and/or expressions were not covered in the period previously stipulated. In this case, studies from 2010 onwards were evaluated. Studies published in English were considered; however, those written in other languages, such as Portuguese, with an abstract in English, were not removed from the archives.

## 2. Relationship between Disease Occurrence and Type of Donkey and Mule Farming

### 2.1. Overview of Donkey and Mule Farming in Brazil

Donkeys and mules represent a growing source of income, especially in Brazil, which has approximately 1 million donkeys [2,4]. However, although there is a potential consumer market, the Brazilian market is still not very expressive, which may be linked to the gradually decreasing number of these animals over the years, a decline that reached 37.08% in 2016 [5].

Another factor to consider is the local culture, which associates the use of equids mostly for traction and other field activities. Despite the economic importance, the exact number of donkeys and mules is uncertain, partially due to extensive farming and absence of technical guidelines, and cultural aspects that associate these animals to poverty, generating abandonment resulting in traffic accidents in the region [5,13].

Despite similarities, donkeys and mules are distinct animals that originated from the cross-breeding of *Equus asinus* and *Equus caballus* species [14]. These animals have intermediate characteristics between the progenitor species making their rearing relatively easy due to their rusticity, easy breeding, longevity, less selective eating habits, docile temperament, and dexterity in performing agricultural activities. In Brazil, three types of donkeys which originated from Europe and Africa are recognized; the northeastern donkey is well adapted to the semiarid climate [5]. Donkeys and mules are part of the herd in all Brazilian states, which may be linked to their use in the colonial period while expanding routes in the interior of the country.

The identification of these characteristics favored the investment in research directed to the biotechnology of reproduction, serological research of microorganisms, as well as in the food sector, raising the topic of the welfare of these animals. In the state of Bahia, Brazil, a private company producing donkey meat and donkey-hide gelatin became an important exporter to China [4,5]. The significant increase in the export of donkey hide to China [4].

Similarly to ruminants, the erroneous notion that donkeys and mules are resistant to the environment and adverse conditions is disseminated among farmers, generating carelessness with nutritional and sanitary management, it is not uncommon to observe working animals with chronic disease and skin lacerations due to whipping [13,15]. The visibility that the articles achieve is essential to raise awareness and disseminate technical information. In this context, Non-Governmental Organizations and other entities commonly promote care in the farming of these animals, although government agencies take time to follow their recommendations [16].

There is further a growing number of articles published in journals relevant to the scientific community addressing the health of these animals. A previous study tracking publications on donkeys [13] showed that until 2018, there were only 114 publications from 56 different countries discussing varying topics. In this study, the themes of reproduction followed by studies on anatomy, pathology, surgery, and equine medicine were highlighted. This shows, among other things, that there is still a vast field of research to be explored with donkeys and mules.

### 2.2. Disease Profile of Donkeys and Mules

Undoubtedly, diseases are the main obstacle in animal husbandry, so much so that occurrence of disease outbreaks can generate significant losses. The role of diagnostic laboratories is essential, considering that infectious diseases stand out among all other diseases that affect herbivores, and are generally due to digestive disorders [17,18,19].

Even considering the economic relevance, the diagnosis of equine diseases is still precarious in the literature. Diseases in donkeys and mules are largely unknown [20]. Generally, these animals develop diseases similar to horses, however, certain irregularities must be considered before a presumptive diagnosis. The clinical differences tend to be quite subtle, such as demonstration of pain, which is generally not as expressive as in horses, justifying differentiated physical examination [14].

Therefore, studies aimed at diagnosing these diseases should be stimulated. The importance of these studies is even more relevant when considering zoonotic agents, such as the West Nile virus, a micro-organism that has re-emerged in recent years and that leads to severe neurological lesions in horses and humans [21]. A serological investigation was performed on donkeys and mules in southern Spain after the detection of West Nile virus infection in the region. It was observed that nine of the 90 herds evaluated contained at least one seropositive animal and antibodies against the virus were further detected in 1/4 of the donkeys coming from farms where the cases were confirmed in horses, demonstrating that serological surveillance of sentinel donkeys and mules is necessary for the epidemiological monitoring of these diseases [8].

In Brazil, a recent study demonstrated the frequency of antibodies to some equine diseases in donkeys in the state of São Paulo. Of the 85 serums evaluated, it was estimated that 50.6% exhibited antibodies against the H3N8 subtype of the influenza virus, 47% against the equine Herpesvirus, and 20% against the equine arteritis virus, demonstrating that these agents circulate among donkeys in the region and reiterating the importance of epidemiological monitoring of equids in Brazil [7].

It is important to highlight, however, that a significant portion of diseases diagnosed in domestic animals is performed through necropsy examination. Many retrospective studies include pathological results and estimate the frequency of the diseases in a certain geographical region, thus improving local clinical diagnosis, as it is possible to draw an epidemiological and clinical-pathological profile in most of these studies. In Brazil, only some studies approach the main equine diseases [17,22,23]. However, the literature is limited regarding equine diseases, with only two studies [11,12] conducted in the Brazilian semiarid region being found.

A North American study on causes of death and reason for euthanasia in equids highlighted digestive disorders and lesions in the pituitary gland and locomotor system. This study focused especially on geriatric diseases, represented by neurological, urinary, and neoplastic disorders, which alone accounted for more than 18% of the causes of death in all animals evaluated [24]. These results are important when considering that donkeys and mules have great longevity and often need appropriate veterinary medical attention.

### 2.3. Skin Diseases Diagnosed in Donkeys and Mules

Skin lesions have been reported in horses in several regions of the world, mainly as case reports with the occasional retrospective study. Regarding skin diseases, there is variation in the origin of the lesions, suggesting individual differences of the species or factors related to the environment, for example traumatic injuries and lesions. Infectious and parasitic dermatopathies impact the quality of life and skin byproducts of these animals. Thus, certain studies report the occurrence or frequency of integument pathologies that may interfere with donkey and mule skin health.

As for infectious and parasitic diseases, the first report of skin besnoitiosis by *Besnoitia besnnetti* in donkeys in the United Kingdom was recently published, referring to the analysis of 20 tissue samples characterized by nodular lesions on skin, mucous membranes, and muco-cutaneous transitions [25]. A very important issue in this study was the fact that the lesions were frequently attributed to sarcoid when macroscopically evaluated, the histopathological findings essential for the definitive diagnosis of a disease that had never before been diagnosed in the country, showing the need to pay more attention to routine cases for skin and mucous membrane lesions in donkeys and mules.

These studies on skin diseases further the understanding of the epidemiology and clinical presentation of diseases and infer the prognosis of the patients. A study conducted in Egypt to evaluate filarial infection in donkeys showed a significant number of filarial lesions in a period of two years, which were not restricted only to the integument. Of the 188 animals studied, 163 were parasitized by *Onchocerca cervicalis,* followed by *Setaria equina*, *Parafilaria multipapillosa*, and *Onchocerca reticulata* being the infection most common in adult males aged five to fifteen years [26].

A retrospective study on skin diseases in donkeys from European countries and the USA has recently been published, emphasizing the geographical distribution of dermatopathies on donkeys in the region. In this study, the following diseases were highlighted: hypersensitivity to insect bites, sarcoid, habronemiasis, superficial pyoderma, and dermatophytosis, among other less expressive diseases. Dermatopathies were common pathologies in equids and, as was already expected, factors such as age, sex, and diagnosis varied by geographical location, showing the importance of a thorough dermatological examination, regardless of the reason and clinical presentation of these diseases [27].

The following tables (Table 1 and Table 2) show some of the main skin disorders caused by fungi, bacteria, parasites and oomycetes described in donkeys and mules in the veterinary literature.

Ulcerative lymphangitis is caused by bacteria belonging to the genera *Corynebacterium pseudotuberculosis*, *Staphylococcus* spp., *Streptococcus* spp., *Pseudomonas aeruginosa* and *Rhodococcus equi*. They initially occur as swelling that fistulates and drains purulent content, progressing to cutaneous or subcutaneous abscesses accompanied by edema. Lesions can be seen particularly in the limbs, such as hocks and fetlock, usually accompanying the lymphatic chain [39,40,41]. As for the ectoparasites that affect the skin of donkeys and mules, there are different agents involved such as diptera causing myiasis, lice and scabies.

*Dermatobia hominis*, *Gasterophilus nasalis,* and *Oestrus ovis* are examples of myiasis-causing flies. Infestation by small numbers of larvae may have little or no clinical effect on the host. In general, these lesions do not require a biopsy to confirm the diagnosis. Infestations are greater in rainy periods when the proliferation of flies and mosquitoes, however the occurrence of myiasis does not follow seasonality, considering that traumatic injuries and untreated injuries that determine their occurrence. The injuries are variable and depend on the source of the injury [42].

Lice and scabies are frequently observed and their visualization already gives a diagnosis, however the skin may exhibit flaking and hyperemia resulting from the blood meal (sucking lice particularly). The parasites can be observed dispersed in the animal’s coat, usually in those most weakened immunosuppressed. Secondary infections are common, particularly in traumatized skin. The distribution in the animal organism varies according to the species of mite, being *Sarcoptes scabiei* var. *equi* but common in the head and neck; *Psoroptes equi* at the base of the mane and tail hair and *Chorioptes equi* below the hock and knees. *Chorioptes equi* is the most common parasite reported in donkeys and mules, associated with intense itching, which can stimulate the habit of self-mutilation [12,27,28].

Neoplastic diseases are an important group of skin diseases in most animals (Table 3), whether during companionship or reproduction. A study conducted using the records of five North American institutions, concluded that 125 of the 357 donkeys evaluated were diagnosed with neoplasia. The skin tumors stood out, with sarcoid being the most common, followed by soft tissue sarcomas. A very important finding highlighted by the authors was the presence of different neoplasm behavior in donkeys when compared to horses. For example, squamous cell carcinoma and melanoma were considered unusual to rare tumors in donkeys, as well as lymphosarcoma, implying that there may be carcinogenesis differences among equids, which should be considered at the time of diagnosis and therapy, as well as at the time of informing veterinarians of the prevalence of tumors in these species [43].

Skin wounds are a frequent problem in equids, especially those of traumatic origin linked to traction activities.

A study evaluating 148 donkeys subjected to workloads three to five hours a day for three to five days a week in Tanzania showed the presence of wounds (one or more) in 56.1% of the animals. The lesions were mostly on the back and neck, and resulted from contact with harnesses, breast collars, and the cart. According to the authors, the lesions were variable in size and extension, most of them affecting the skin and subcutaneous tissues, some deepening to the musculature, while another portion was associated to granulation tissue, exudation, besides hemorrhages and necrosis [45]. An important finding of this study was the report of owners using substances such as motor oil for the treatment of injuries, a common practice in the northeast of Brazil, attributing to the oil protection and lubrication of the injured skin.

In Ethiopia, of a total of 997 horses, the most common skin diseases included skin wounds, followed by ectoparasites, dermatophilosis, sarcoid, and dermatophytes. A very important finding of this study refers to risk factors for the occurrence of these lesions, with the body score being very important for the occurrence of injuries, as well as what kind of work these animals performed [15]. The use of donkeys and mules for a multitude of tasks in Ethiopia is part of the culture of the country; however, sanitary and nutritional conditions do not always follow the degree of effort to which these animals are subjected to, thus generating the aforementioned injuries. The approach in these cases should be more holistic to consider cultural and socioeconomic issues and, at the same time, to improve the health and welfare of the animals.

The following table (Table 4) show some of the main skin disorders caused by factors related to the environment and traumatic injuries lesions.

Hypersensitivity to mosquito bites in general are progressive lesions starting with papules and crusts, accompanied by alopecia and erythema and may progress to ulceration. Mixed and perivascular dermatitis occurs with hyperplasia, orthokeratosis, crusts, edema, hyperkeratosis in follicles and hyperplasia of adnexal glands [27,28,48]. Typically, the disease manifests itself as chronic and itchy in character, particularly in the periocular region and external auditory canal, extending to the neck, back, abdomen, tail and limbs. Secondary mutilation and infections can occur secondarily and itching is variable, and the exposure of the injured areas to the sun’s rays can cause serious complications [49,50].

Among the autoimmune diseases, pemphigus foliaceus stands out as being progressive and may contain epidermal collarette, papules and crusts, progressing to a scaly and alopecic lesion with or without exudation and easy hair removal. Microscopically these lesions correspond to a dermatitis with intraepidermal pustules with acantholysis and infiltration of intralesional and dermal neutrophils and eosinophils. Affected areas are multifocal that can coalesce and become generalized. They usually start in the face and limbs, but can affect mucocutaneous junctions [27,28,51].

## 3. Equine Dermatopathies in Northeast Brazil

Much of the attention given to donkey skin is due to the commercial value in the international market. In this context, similar to bovine leather, skin lesions may depreciate the final product and interfere with quality. In Brazil however, reports of integument disorders in donkeys and mules are little reported when compared to North American, European, and Asian countries. The low value attributed to these animals in the northeastern rural communities, associated with particularities of the species regarding clinical presentation of some diseases, probably impact the owner’s decision not to take these animals to the veterinarian, resulting in many diseases being underdiagnosed in the region.

A study in the semiarid region of Paraíba State reported that 258 donkeys and mules seen in 10 years presented integumental diseases as the main causes of consultation (88 cases), mainly traumatic wounds, sarcoids, and abscesses in donkeys and traumatic wounds, squamous cell carcinomas, and habronemiasis in mules. A very important finding raised in this study was that most diseases diagnosed could be linked to mistreatment or lack of attention to these animals [11], a factor associated to the cultural habits of many owners.

In the same year, another retrospective study exclusively on equid skin diseases in the semiarid northeast was published [11]. According to this study, only one case of pythiosis was reported in donkeys in the northeast, which was subsequently approached regarding clinical, epidemiological, pathological, immunohistochemical, and molecular aspects [36]. According to the authors, the pattern of the lesion was similar to cases of pythiosis in cattle, and the origin of the disease was attributed to grazing in flooded areas, similarly to what is described in equines.

Other diseases that deserve to be highlighted, despite the less expressive diagnosis, are photosensitizing and allergic diseases, recently diagnosed in donkeys and mules in the northeast of Brazil. A case of allergic dermatitis to a *Culicoides* bite was reported in the state of Pernambuco, characterized by crusted papule and pruritic skin lesions with a clinical progression of two years. A very important finding highlighted in this study was the difficult clinical diagnosis due to similarities with other equid dermatopathies [48].

**Figure 1:**
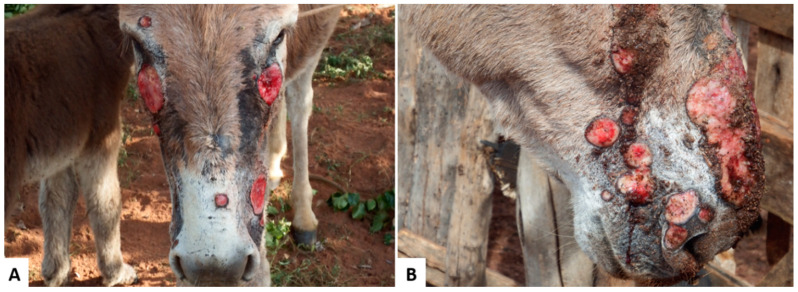
Donkeys naturally poisoned by *Froelichia humboldtiana*. (**A**) Multiple skin ulcers caused by secondary self-mutilation to intense itching; (**B**) Multiple and extensive ulcerated wounds, that drained serous exudate.

Primary photosensitization caused by *Froelichia humboldtiana* in equids have been reported in the semiarid region of Brazil, affecting donkeys and mules. This condition was described as exuberant, ulcerated lesions, with abundant serous exudation and crusts, alopecia, erythema, edema and areas of necrosis (Figure 1), especially in face, croup, and withers, accompanied by intense itching. The lesions were also associated with myiasis and secondary infections and, in many cases, due to the impossibility of treating the wounds, there was high mortality in the herd. The disease occurs at the end of the rainy season in pastures highly invaded by *F. humboldtiana*. Animals usually recover after their removal from areas invaded by this plant [46].

## 4. Conclusions

The study of skin diseases in equines is essential, especially in Brazil, which has an impressive number of donkeys and mules. The evaluation of the clinical and pathological behavior of these diseases implies the implementation of control strategies and the recognition of pathological patterns may be specific to the species.

Geographical variations may further result in significant differences in the prevalence of skin diseases, identifying the need for regional discussions on the emergence of common diseases. In addition, cultural factors may interfere with the frequency of these diseases, which implies losses in animal health and welfare. Incorporating these concepts in an increasingly technological world is perhaps the greatest challenge for the sustainable farming of donkeys and mules.

## Figures and Tables

**Table 1:** Clinical and pathological characteristics of skin diseases caused by fungi and bacteria in donkeys and mules.

Disease	Gross Pathology	Histopathology	Clinical	References
Dermatophilosis (*Dermatophilus congonlensis*)	Lesions are usually crusted, alopecic, circumscribed and with marked agglutination of hair, or spread diffusely.	These areas characterize an exudative dermatitis with the formation of crusts interspersed with layers of exudate.	In general, muzzle, face, eyes, limbs and back are the main affected areas, however they can manifest in a widespread way.	[12,28,29]
Dermatophytosis	Unique, multifocal areas slightly elevated and with regular edges, accompanied by alopecia, flaking and grayish crusts.	This lesion represents hyperplastic dermatitis with suppurative folliculitis, hyperkeratosis, epidermal acanthosis and microabscesses.	The lesions are generally not itchy and start in areas of abrasions with loins, rump and head, but which can expand to the back and flank.	[12,28,29]
Epizootic lymphangitis (*Histoplasma farciminosum*)	Single or multiple nodular areas, of slow growth, which ulcerate and drain purulent content. There is usually granulation tissue surrounding these lesions.	Generally granulomatous lesions with adjacent granulation tissue and intrahistiocytic and extracellular yeasts, stained positively with Grocott’s methenamine silver and Periodic acid-Schiff stains; that can even show budding.	It can occur on lymphatic lines of the legs, neck region, on the skin or in the nasolacrimal region, limbs (in particular after localized trauma).	[27,28,30,31]
Glanders (*Burkholderia mallei*)	Lesions range from nodular swelling in lymph vessels (rosary beads) to abscesses, alopecia, ulcerations and edema.	These lesions are irregular and characterized by a necrosis center surrounded by a granulomatous to pyogranulomatous infiltrate and adjacent fibrous connective tissue.	The nodules usually follow the distribution of the lymphatic vessels, but are observed particularly in the limbs and flank, head and neck, and can “float” on palpation.	[32,33,34]

**Table 2:** Clinical and pathological characteristics of skin diseases caused by parasites and oomycetes in donkeys and mules.

Disease	Gross Pathology	Histopathology	Clinical	References
Habronemiasis (*Habronema* sp.)	Small, crusted nodular lesions, which progressively increase in volume and acquire a spongy and reddish appearance.	These nodulations represent severe eosinophilic dermatitis and panniculitis, with fibroplasia and the presence of intralesional larvae.	It can occur on the skin of the limbs, withers, penis or in the ocular conjunctiva. In conjunctival form it accompanies ocular discharge.	[12,27,29]
Filariosis (*Onchocerca* sp.; *Setaria equina*; *Parafilaria multipapillosa*)	Nodular swelling, of variable size, which may ulcerate.	These lesions correspond to a granulomatous inflammation that usually forms in response to the larvae.	Skin infections are often associated with *Oncocerca cervicalis*, in the nuchal ligament but can affect tendons and ligaments of the limbs.	[26,28,35]
Pythiosis (*Pythium insidiosum*)	Ulcerated nodules and drain sero-bloody secretion. Accompanied by fibrous, whitish and shiny fabric, interspersed by kunkers.	Areas of necrosis and eosinophilic infiltrate, surrounded by granulation tissue and fibrosis, with negative images of intralesional hyphae; Grocott-Gomori methenamine silver stain positive.	Lesions are seen in the limbs, ventral abdominal region, chest, neck, face, lips, breast and genitals, and can be itchy, predisposing to self-mutilation.	[12,28,36,37,38]
Besnoitiosis (*Besnoitia* spp.)	They may appear as small, multiple, round, yellowish-white, punctate lesions, with thickening, peeling, formation of wrinkles/folds and lichenification.	These lesions represent a mixed inflammatory infiltrate involving the cysts of *Besnoitia* spp. In addition, hypeceratosis, scales and crusts can be observed accompanying dermatitis.	Lesions can be seen in the neck, head, limbs and perineum are particularly affected. The lesions can also appear in areas that have suffered previous trauma or in self-inflicted trauma.	[25,27]

**Table 3:** Clinical and pathological characteristics of the main primary cutaneous neoplasms diagnosed in donkeys and mules.

Disease	Gross Pathology	Histopathology	Clinical	References
Sarcoids	Nodulations are classified based on morphological patterns, being of the type, fibroblastic, verrucous and mixed.	These lesions are characterized as proliferative masses that have two histological constituents: an epithelial tissue and a dermal connective tissue with marked disorganized proliferation of connective tissue.	Commonly affected sites include ears, labial commissures, ventral trunk and feet.	[43,44]
Squamous cell carcinoma	The lesions are nodular, expansive, usually firm and sessile, which gradually increase in volume, ulcerating, with easy bleeding and crusting.	These nodulations correspond to infiltrative and irregular masses with cells arranged in cords or nests whose center may contain aggregates of keratin (corneal pearls) according to the degree of differentiation.	Its occurrence is often attributed to sun exposure in anatomical regions unprotected from pigmentation or hair, particularly in eyelids, ears, snout, perineum and udder.	[12]
Papilloma	The lesions are often arborescent or filiform, with a dry surface and that detach from the skin under traction.	This proliferation is benign and corresponds to an epithelial hyperplasia, forming papillary projections, with moderate supporting connective tissue.	The anatomical location is variable but can be seen particularly in the head, neck, belly, limbs, face, penis and base of the tail. They are usually benign and self-limiting.	[12,43]
Melanoma	Melanocytic tumors occur as single or multiple, shiny masses, usually blackened and multilobulated, with a high rate of metastasis.	Neoplastic melanocytes can exhibit intense pleomorphism with high mitotic activity, and are arranged in nests.	They are commonly seen in old, light-haired equines, particularly in the perineum, base of the tail and external genitalia.	[12,44]

**Table 4:** Environmental disease described in donkeys and mules.

Disease	Gross Pathology	Histopathology	Clinical	References
Photosensitization	The lesions are characterized by erosions and crusts accompanied by hyperemia, serous exudate, and, subsequently, to cracks and cutaneous detachment.	Microscopically, there are hyperkeratosis, ulcers in the epidermis, crusting and infiltration in the dermis that varies from polymorphonuclear to mononuclear cells.	It particularly affects depigmented areas such as the snout, udder, back and vulva. This condition can occur primary or secondary. The animal exhibits intense itching, contributing to self-mutilation.	[46,47]
Wounds, exuberant granulation tissue	Initial wounds can be of varied causes, leading to ulcerative and crusted lesions. These lesions can evolve into the exuberant granulation tissue becoming spongy, irregular, with no evident exudation.	In these lesions, there is a marked proliferation of fibrous connective tissue, neovascularization, fibroplasia, in addition to a chronic active inflammatory infiltrate, depending on whether the pathogenic stimulus persists or not.	Varied location, usually associated with the use of ropes, saddles and whips for containment, being commonly observed in the neck, limbs, back and tail.	[12,28,45]

## References

[1] The Food and Agriculture Organization-FAO (2016). http://www.fao.org/faostat/en/#home.

[2] The Food and Agriculture Organization-FAO (2018). http://www.fao.org/faostat/en/#data/QA.

[3] Luís C., Bastos-Silveira C., Cothran E.G., Oom M.D.M. (2006). Iberian origins of New World horse breeds. J. Hered..

[4] Baker M. (2017). Sob a Pele-O Comércio Emergente de Pele de Asno e Suas Implicações Para o Bem-Estar e os Meios de Subsistência dos Asnos.

[5] Carneiro G.F., Cavalcante Lucena J.E., de Oliveira Barros L. (2018). The current situation and trend of the donkey industry in South America. J. Equine Vet. Sci..

[6] Köhle N., Smith C.A., Kohle N., Jaivin L. (2018). Feasting on Donkey Skin. Conspicuous Consumption.

[7] Lara M.C.C.S.H., Villalobos E.M.C., Cunha S.E.M., Oliveira J.V., Castro C., Nassar A.F.C., Silva L.M.P., Okuda L.H., Romaldini A.H.C.N., Cunha M.S. (2017). Occurrence of viral diseases in donkeys (*Equus asinus*) in São Paulo State, Brazil. Braz. J. Vet. Res. An. Sci..

[8] García-Bocanegra I., Arenas-Montes A., Jaén-Téllez J.A., Napp S., Fernández-Morente M., Arenas A. (2012). Use of sentinel serosurveillance of mules and donkeys in the monitoring of West Nile virus infection. Vet. J..

[9] Lima T.S. (2019). Caracterização Clínico-patológica e Epidemiológica das Dermatopatias de Ruminantes no Agreste da Paraíba. Master’s Dissertation.

[10] Assis-Brasil N.D., Marcolongo-Pereira C., Stigger A.L., Fiss L., Santos B.L., Coelho A.C.B., Sallis E.S.V., Fernandes C.G., Schild A.L. (2015). Dermatopatias em equinos na região sul do Rio Grande do Sul: Estudo de 710 casos. Ciên. Rural..

[11] Pessoa A.F.A., Pessoa C.R.M., Miranda Neto E.G., Riet-Correa F. (2014). Doenças de asininos e muares no semiárido brasileiro. Pesq. Vet. Bras..

[12] Pessoa A.F.A., Pessoa C.R.M., Miranda Neto E.G., Dantas A.F.M., Riet-Correa F. (2014). Doenças de pele em equídeos no semiárido brasileiro. Pesq. Vet. Bras..

[13] McLean A.K., Navas Gonzalez F.J. (2018). Can scientists influence donkey welfare? Historical perspective and a contemporary view. J. Equine Vet. Sci..

[14] Miranda A.L.S., Palhares M.S. (2017). Muares: Características, origem e particularidades clínico-laboratoriais. Rev. Cient. Med. Vet..

[15] Tesfaye A., Tekle Y., Taddele H., Gezahagn K., Yihdego H. (2015). Survey of Common Skin Problem of Working Equines in and Around Mekelle, North Ethiopia. Acad. J. Anim. Dis..

[16] Davis E. (2019). Donkey and Mule Welfare. Vet. Clin. N. Am. Equine Pract..

[17] Pierezan F., Rissi D.R., Rech R.R., Fighera R.A., Brum J.S., Barros C.S.L. (2009). Achados de necropsia relacionados com a morte de 335 eqüinos: 1968–2007. Pesq. Vet. Bras..

[18] Lucena R.B., Pierezan F., Kommers G.D., Irigoyen L.F., Fighera R.A., Barros C.S.L. (2010). Doenças de bovinos no Sul do Brasil: 6.706 casos. Pesq. Vet. Bras..

[19] Marques A.L.A., Aguiar G.M.N., Lira M.A.A., Miranda Neto E.G., Azevedo S.S., Simões S.V.D. (2018). Enfermidades do sistema digestório de bovinos da região semiárida do Brasil. Pesq. Vet. Bras..

[20] Barrandeguy M.E., Carossino M. (2018). Infectious diseases in donkeys and mules: An overview and update. J. Equine Vet. Sci..

[21] Mavrouli M., Vrioni G., Kapsimali V., Tsiamis C., Mavroulis S., Pervanidou D., Billinis C., Hadjichristodoulou C., Tsakris A. (2019). Reemergence of West Nile Virus Infections in Southern Greece, 2017. Am. J. Trop. Med. Hyg..

[22] Souza T.M., Brum J.S., Fighera R.A., Brass K.E., Barros C.S.L. (2011). Prevalência dos tumores cutâneos de equinos diagnosticados no Laboratório de Patologia Veterinária da Universidade Federal de Santa Maria, Rio Grande do Sul. Pesq. Vet. Bras..

[23] Marcolongo-Pereira C., Estima-Silva P., Soares M.P., Sallis E.S.V., Grecco F.B., Fernandes C.G., Raffi M.B., Schild A.L. (2014). Doenças de equinos na região Sul do Rio Grande do Sul. Pesq. Vet. Bras..

[24] Miller M.A., Moore G.E., Bertin F.R., Kritchevsky J.E. (2015). What’s New in Old Horses? Postmortem Diagnoses in Mature and Aged Equids. Vet. Pathol..

[25] Elsheikha H.M., Schares G., Paraschou G., Sullivan R., Fox R. (2020). First record of besnoitiosis caused by *Besnoitia bennetti* in donkeys from the UK. Parasites Vectors.

[26] Radwan A.M., Ahmed N.E., Elakabawy L.M., Ramadan M.Y., Elmadawy R.S. (2016). Prevalence and pathogenesis of some filarial nematodes infecting donkeys in Egypt. Vet. World.

[27] White S.D., Bourdeau P.J., Brement T., Vandenabeele S.I., Haspeslagh M., Bruet V., Van Oldruitenborgh-Oosterbaan M.M.S. (2019). Skin disease in donkeys (*Equus asinus)*: A retrospective study from four veterinary schools. Vet. Dermatol..

[28] Knottenbelt D.C. (2019). Skin Disorders of the Donkey and Mule. Vet. Clin Equine..

[29] Alvarez J.A.C., Socarras T.O., Tous M.G. (2017). Dermopatias en burros de trabajo (*Equus asinus*) en areas rurales de Cordoba (Colombia). Rev. Med. Vet..

[30] Meselu D., Abebe R., Mekibib B. (2018). Prevalence of Epizootic Lymphangitis and bodily distribuition of lesions in cart-mules in Behair Dar Town, Northwest Ethiopia. J. Vet. Sci. Technol..

[31] Mahendra P. (2019). Occurrence of Cutaneous Epizootic Lymphangitis in Working Donkeys in Debre Zeit, Ethiopia. EC Microbiol..

[32] Mota R.A., Oliveira A.A.F., Pinheiro Junior J.W., Silva L.B.G., Brito M.F., Rabelo S.S.A. (2010). Glanders in donkeys (*Equus asinus*) in the state of Pernambuco, Brazil: A case report. Braz. J. Microbiol..

[33] Rabelo S.S.A., Soares P.C., Silva L.B.G., Cunha A.P., Nascimento Sobrinho E., Pinheiro Junior J.W., Barbosa M.A.G., Mota R.A. (2006). Indicadores Clínicos em Muares Naturalmente Infectados pela *Burkholderia mallei*. Vet. Zootec..

[34] Mota R.A., Brito M.F., Castro F.J.C., Massa M. (2000). Mormo em eqüídeos nos estados de Pernambuco e Alagoas. Pesq. Vet. Bras..

[35] Ghahvei Y., Mirzaei M., Hashemnia S., Golchin M., Kheirandish R., Uni S., Endoza-Roldan J.A., Otranto D., Sazmand A. (2020). Scanning electron microscopy of *Onchocerca fasciata* (Filarioidea: Onchocercidae) adults, microfilariae and eggs with notes on histopathological findings in camels. Parasit. Vectors.

[36] Maia L.A., Olinda R.G., Araújo T.F., Firmino P.R., Nakazato L., Miranda Neto E.G., Riet-Correa F., Dantas A.F.M. (2016). Cutaneous pythiosis in a donkey (*Equus asinus*) in Brazil. J. Vet. Diagn. Invest..

[37] Tabosa I.M., Medeiros V.T., Dantas A.F.M., Azevedo E.O., Maia J.C. (1999). Pitiose cutânea em equinos no semi-árido da Paraíba. ABMVZ.

[38] Santos C.E.P., Santurio J.M., Colodel E.M., Juliano R.S., Silva J.A., Marques L.C. (2011). Contribuição ao Estudo da Pitose Cutânea em Equídeos do Pantanal Norte, Brasil. ARS Vet..

[39] Leite N.M., Rocha M.V., Souza K.M., Vago P.B. (2019). Linfangite ulcerativa em equino: Relato de caso. PUBVET.

[40] Sureshjani M.H., Atyabi N., Tazikeh A., Falahatipour S.K., Hashemian M. (2014). Isolation of Rhodococcus equi from a mule with cutaneous wound. Comp. Clin. Pathol..

[41] Prescott J.F. (1991). Rhodococcus equi: An animal and human pathogen. Clin. Microbiol. Rev..

[42] Guimarães J.H., Papavero N., Prado A.P. (1982). As miíases na região neotropital (identificação, biologia, bibliografia). Rev. Bras. Zool..

[43] Davis C.R., Valentine B.A., Gordon E., McDonough S.P., Schaffer P.A., Allen A.L., Pesavento P. (2016). Neoplasia in 125 donkeys (*Equus asinus*): Literature review and a survey of five veterinary schools in the United States and Canada. J. Vet. Diagn. Investig..

[44] Alberti T.S., Zamboni R., Venancio F.R., Scheid H.V., Bermann C.S., Raffi M.B., Sallis E.S.V. (2019). Melanoma anaplásico em equino de pelagem tordilha com metástase em osso e músculo. Cien. Anim..

[45] Rayner E., Airikkala-Otter I., Susheelan A., Gibson A., Itaba R., Mayani T., Mellanby R.J., Gamble L. (2019). Prevalence of skin wounds in working donkeys in Bukombe, Tanzania. Vet. Rec..

[46] Knupp S.N.R., Knupp L.S., Riet-Correa F., Lucena R.B. (2016). Plants that cause photosensitivity in ruminants in Brazil. Semin. Ciênc. Agrár..

[47] Amado G.P., Silva C.C.B., Barbosa F.M.S., Nascimento H.H.L., Malta K.C., Azevedo M.V., Lacerda-Lucena P.B., Lucena R.B. (2018). Surtos de fotossensibilização e dermatite alérgica em ruminantes e equídeos no Nordeste do Brasil. Pesq. Vet. Bras..

[48] Silva T.I.B., Melchior L.A.K., Baptista Filho L.C.F., Fernandes A.C.C., Silva L.G., Vasconcelos K.F., Revorêdo R.G., Silva D.D., Melo L.E.H. (2017). Dermatite alérgica à picada de Culicoides em muar: Relato de caso. Arq. Bras. Med. Vet. Zootec..

[49] Schaffartzik A., Hamza E., Janda J., Crameri R., Marti E., Rhyner C. (2012). Equine insect bite hypersensitivity: What do we know?. Vet Immunol. Immunopathol..

[50] Corrêa T.G., Ferreira J.M., Riet-Correa G., Ruas J.L., Schild A.L., Riet-Correa F., Guimarães A., Felippe-Bauer M.L. (2007). Seasonal allergic dermatitis in sheep in southern Brazil caused by *Culicoides insignis* (Diptera: Ceratopogonidae). Vet. Parasitol..

[51] Monteiro G.A., Souza M.V., Conceição L.G., Balbi C.L., Borba R., Moreira M.A.S. (2007). Pênfigo foliáceo em um equino. Ciência Rural.

